# Muometric positioning system (muPS) utilizing direction vectors of cosmic-ray muons for wireless indoor navigation at a centimeter-level accuracy

**DOI:** 10.1038/s41598-023-41910-y

**Published:** 2023-09-15

**Authors:** Hiroyuki K. M. Tanaka

**Affiliations:** 1 University of Tokyo, Tokyo, Japan; 2 International Virtual Muography Institute (VMI), Global, Tokyo, Japan

**Keywords:** Particle physics, Experimental particle physics, Aerospace engineering, Civil engineering, Natural hazards, Solid Earth sciences, Engineering

## Abstract

Since the development of many future technologies are becoming more and more dependent on indoor navigation, various alternative navigation techniques have been proposed with radio waves, acoustic, and laser beam signals. In 2020, muometric positioning system (muPS) was proposed as a new indoor navigation technique; in 2022, the first prototype of wireless muPS was demonstrated in underground environments. However, in this first physical demonstration, its navigation accuracy was limited to 2–14 m which is far from the level required for the practical indoor navigation applications. This positioning error was an intrinsic problem associated with the clock that was used for determining the time of flight (ToF) of the muons, and it was practically impossible to attain cm-level accuracy with this initial approach. This paper introduces the completely new positioning concept for muPS, Vector muPS, which works by determining direction vectors of incoming muons instead of utilizing ToF. It is relatively easier to attain a 10-mrad level angular resolution with muon trackers that have been used for muographic imagery. Therefore, Vector muPS retains the unique capacity to operate wirelessly in indoor environments and also has the capacity to achieve a cm-level accuracy. By utilizing an essentially different concept from what is used in other navigation techniques, (measuring the distance between the reference and the receiver), Vector muPS enables more flexible, and longer-term stable positioning. Anticipated applications and the future outlook of Vector muPS is also discussed.

## Introduction

Indoor navigation systems can serve many practical purposes including for human transportation navigation guidance systems, pinpointing the location of a missing person for emergency rescue and automated robot operation in factories as well as navigation through mines and underground facilities^1,2^. Thus far, several indoor positioning/navigation techniques have been proposed for use in regions where more standard navigation techniques like global positioning system (GPS) or other global navigation satellite systems are unavailable. These techniques have different navigation ranges and navigation accuracies. While the ZigBee technique^3^ and the radio frequency identification (RFID) technique^3^ have a navigation range up to ~ 100 m, their navigation accuracy is limited to ≥ 10 m. On the other hand, the light detection and ranging (LiDAR) technique^4^ and the acoustic technique^5^ have much shorter navigation range of up to ~ 10 m, but a cm-level navigation accuracy is achievable. These techniques share the same basic concept: “geometric” relations between the receiver and the references (signal transmitters or reference objects around the receiver). However, there are 2 main problems hampering these techniques:

1. Unlike outdoor environments, indoor areas usually contain a number of obstacles, many of which increase the difficulty of implementing navigation systems. Many common indoor obstacles easily interfere with the signals (radio waves, sound, or a laser beam, for instance) used for these techniques.
2. Additionally, obstacles often negatively affect the accuracy and range of these techniques which have a tendency to cause the receiver to have multipath conditions and which can cause errors^6^.

Recent developments in Wi-Fi Fingerprinting^7^, Ultra-Wideband (UWB)^8^, and Bluetooth Low Energy (BLE)^9^ techniques have significantly improved positioning accuracy. More recently, Marvelmind Indoor “GPS”^10^, showed its performance that enabled a positioning accuracy of ± 2 cm which offers a GPS-RTK level positioning accuracy^11^. However, these techniques all use radio waves and thus, positioning signals are influenced by the metal-made obstacles located between the beacons and clients. muPS was invented to address this problem and to operate in situations when radio waves are highly influenced by obstacles or they cannot be used.

Cosmic-ray muons are generated in the atmosphere as secondary cosmic rays; they have ubiquitous and universal characteristics, meaning cosmic-ray muons can be utilized worldwide on the Earth’s surface as well as in indoor, subterranean and underwater regions. The majority of cosmic-ray muons are relativistic, traveling at almost the speed of light in vacuum through any kind of material and their speed is not affected by the media condition they travel through. Since muons are 205 times heavier than electrons, their radiative process in matter is strongly suppressed and consequently, relativistic muons have a strong penetration capability with a virtually straight trajectory. These specific features of relativistic cosmic-ray muons have been applied to imaging of gigantic objects^12,13^, time synchronization^14–16^, cryptographic communication^17,18^, and navigation^19–21^.

In 2020, the muPS (muometric positioning system), being the first cosmic-ray-muon-based three-dimensional positioning technique, was proposed by Tanaka^19^ and formed the basic concept for several subsidiary muometric navigation techniques. By taking advantage of the penetration capability, the constant speed, and the straight trajectories (virtually unchanged by the type of matter traversed) of high energy cosmic-ray muons, the position of the receiver detector can be accurately determined within the coordinate defined by the reference detectors. In the 2020 lab-scale experiment, since these reference detectors and the receiver detector were connected via wires, the time synchronization between these detectors was trivial and cm-level positioning accuracy was attainable. However, this wired system substantially limited the flexibility that most navigation situations would require. Accordingly, a new wireless muPS system called Muometric Wireless Navigation System (MuWNS) was proposed in 2022 to function without the requirement of a wired configuration between the reference detectors and the receiver detector^20^. Eventually, MuWNS was demonstrated for the first time in a physical environment (underground environment) to show its capability to enable indoor navigation with an accuracy of 2–14 m^21^. However, this positioning accuracy was far from the accuracy required for practical applications such as automation of factory operations which requires ~ cm-level accuracy. This positioning accuracy simply depends on the wireless synchronization stability, and in order to attain the positioning accuracy of < 10 cm, the temporal fluctuations in local clocks associated with detectors must be suppressed below 300 ps.

The concept of MuWNS was developed with the objective of having an energy efficient/relatively inexpensive alternative method for pinpointing the position of a receiver even in situations where physical obstacles would hinder the operation/accuracy of other available techniques. MuWNS^20,21^ relies on the time of flight (TOF) of naturally occurring muons as positioning markers; muon detection events between two or more reference and receiver detectors within a narrow time window are recognized as being the same muons, and once marked, these can then be used to calculate the TOF; hence, this can determine the distance between the reference and receiver detectors. MuWNS requires very precise time synchronization between the reference detectors and the receiver detector since cosmic-ray muons travel ~ 30 cm in 1 ns. Since a wireless sub-ns time synchronization technique does not yet exist, precise but inexpensive local clocks called an oven-controlled crystal oscillators (OCXO) have been used^21^. However, the frequency of these local clocks tends to fluctuate by tens of nanoseconds, leading to an uncertainty of more than a few m in positioning^21^.

With Vector muPS, instead of relying on extremely accurate timing for identifying identical muon events, the muon's direction vectors, i.e., the muon's incident angles are accurately measured both with the reference detector and the receiver detector; if the angles between the 2 detectors match within a specified narrow angular window, these events are identified and marked as the same muon events. This technique is possible because the direction of muons is virtually unaltered by the matter they traverse. While RF signals or a laser beam cannot penetrate a 5-mm thick plate of Fe, cosmic-ray muons can easily penetrate this Fe plate and the RMS scattering angle of 3-GeV muons (a typical energy of cosmic-ray muons) is only 1.8 mrad. The advantage of the Vector muPS system is that ~ cm level positioning accuracy is possible without incurring the costly and challenging process of maintaining the < 300 ps timing accuracy required for MuWNS. By removing the local clocks that are required to have cesium-oscillator or active-hydrogen-maser level accuracy from MuWNS, this direction-vector measuring strategy makes Vector muPS low cost, compact, and stable; hence adaptable to a wider range of applications. On the other hand, the main challenge of Vector muPS is that the angular relationship between the reference detector and the receiver detector must be accurately and frequently monitored. However, as will be discussed later, overcoming this drawback is possible by incorporating relatively cheap and widely-available inclinometers (instead of the expensive precision clocks required for MuWNS) into the receiver detector. In this paper, the principle of Vector muPS and simulation results for possible applicable environments are introduced, and applications of Vector muPS are discussed. The current paper first introduces the Principle of Vector muPS, and subsequently the expected positioning frequency (signal update rate), accuracy, and noise will be discussed. Then simulation results are discussed based on the practical muPS device deployment models. Finally, applications of this technique are introduced in order to discuss the usability of this technique.

## Results

### Principle of Vector muPS

**Figure 1:**
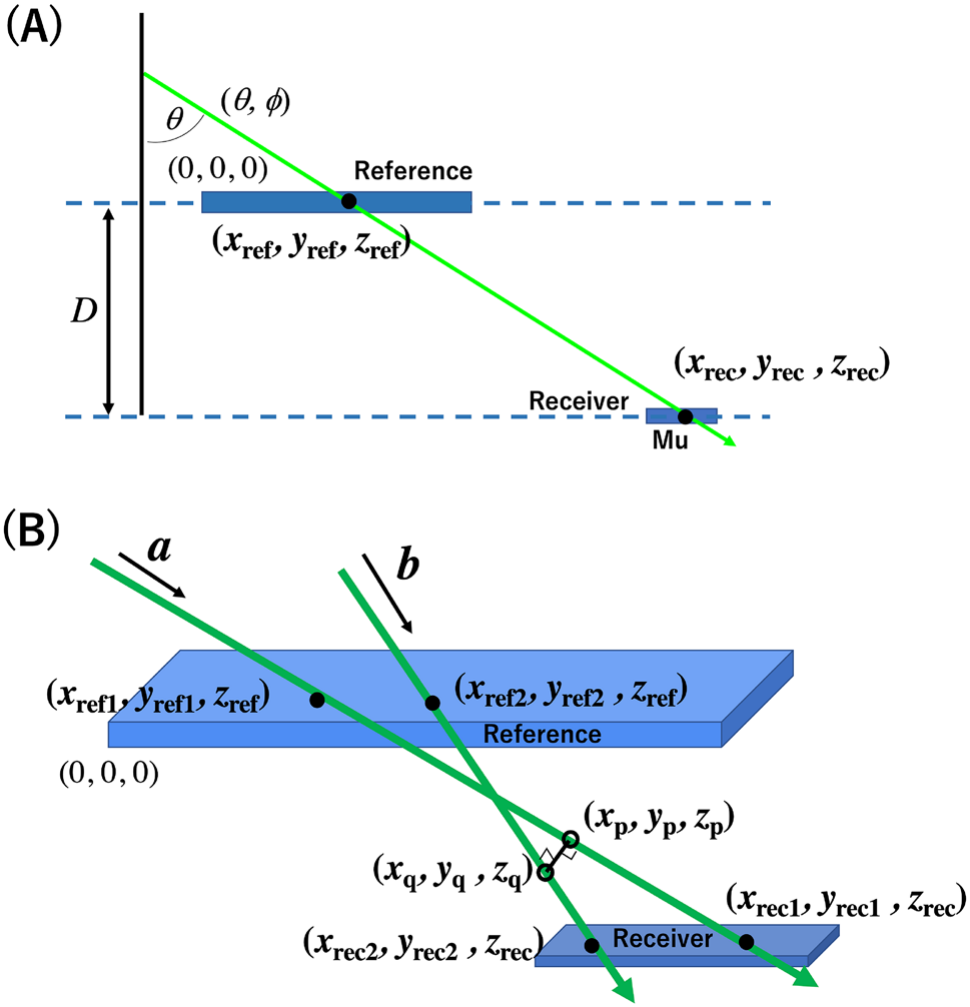
Scheme of Vector muPS positioning. The scheme used when the vertical position of the receiver is known (**A**), and the scheme used when the vertical position of the receiver is not known (**B**) are shown. The notations of ***a***, ***b***, (*x*_ref1_, *y*_ref1_, *z*_ref_), (*x*_ref2_, *y*_ref2_, *z*_ref_), (*x*_rec1_, *y*_rec1_, *z*_rec_), (*x*_rec2_, *y*_rec2_, *z*_rec_), (*x*_p_*, y*_p_*, z*_p_), and (*x*_q_*, y*_q_*, z*_q_) are given in the main text.

Unlike MuWNS, with Vector muPS, the position of the receiver detector is determined by the angle of the incident muons. If the vertical position (*z* = *D*) is already known or given by an external source, only one muon track is sufficient to pin point the muon hitting positions on the receiver detector (*x*_rec_*, y*_rec_*, z*_rec_) such that:

1 $$\begin{aligned} \left( {x_{{{\text{rec}}}} , \, y_{{{\text{rec}}}} , \, z_{{{\text{rec}}}} } \right) & = \left( {x_{{{\text{ref}}}} + (z_{{{\text{rec}}}} - \, z_{{{\text{ref}}}} )\,{\text{tan}}\theta , \, y_{{{\text{ref}}}} + (z_{{{\text{rec}}}} - \, z_{{{\text{ref}}}} ){\text{tan}}\theta \;{\text{tan}}\phi ,(z_{{{\text{rec}}}} - \, z_{{{\text{ref}}}} )} \right) \\ & = \, \left( {x_{{{\text{ref}}}} + D\,{\text{tan}}\theta , \, y_{{{\text{ref}}}} + D\,{\text{tan}}\theta \;{\text{tan}}\phi ,D} \right), \\ \end{aligned}$$

where (*x*_ref_, *y*_ref_, *z*_ref_) is the muon hitting positions on the reference detector. *θ* and *ϕ* are the zenith angle and azimuth angle of the incident muons (Fig. 1A).

When the vertical position of the receiver is not known, we additionally define (*x*_ref2_, *y*_ref2_, *z*_ref_) and (*x*_rec2_*, y*_rec2_*, z*_rec_) respectively as the intersection point between the second muon trajectory and the reference detector, and that between the second muon trajectory and the receiver detector. As shown below, if (*x*_rec1_*, y*_rec1_*, z*_rec_) and (*x*_rec2_*, y*_rec2_*, z*_rec_) are located closer than the required positioning resolution, the three-dimensional position of the receiver detector can be derived by two muon trajectories since it is reasonable to approximate that the position where these two trajectories approach closest is the position of the receiver (Fig. 1B).

By respectively introducing the vectors ***a*** (*a*_*x*_, *a*_*y*_, *a*_*z*_) and ***b*** (*b*_*x*_, *b*_*y*_, *b*_*z*_), and two parameters *s* and *t* for Trajectory A and Trajectory B, we define the three-dimensional positions (*x*_p_*, y*_p_*, z*_p_) and (*x*_q_*, y*_q_*, z*_q_) respectively on Trajectory A and Trajectory B where these 2 trajectories approach the closest such that:

2-1 $$\left( {x_{{\text{p}}} , \, y_{{\text{p}}} , \, z_{{\text{p}}} } \right) = \left( {x_{{{\text{ref1}}}} + sa_{x} , \, y_{{{\text{ref1}}}} + sa_{y} , \, z_{{{\text{ref}}}} + sa_{z} } \right),$$

2-2 $$\left( {x_{{\text{q}}} , \, y_{{\text{q}}} , \, z_{{\text{q}}} } \right) = \left( {x_{{{\text{ref2}}}} + tb_{x} , \, y_{{{\text{ref2}}}} + tb_{y} , \, z_{{{\text{ref}}}} + tb_{z} } \right),$$

We define *d*^2^ as the squared distance between the closest points on Trajectory A and Trajectory B such that:

3 $$d^{{2}} = \left( {x_{{\text{p}}} - x_{{\text{q}}} } \right)^{{2}} + \left( {y_{{\text{p}}} - y_{{\text{q}}} } \right)^{{2}} + \left( {z_{{\text{p}}} - z_{{\text{q}}} } \right)^{{2}} .$$

Since (*x*_ref1_*, y*_ref1_*, z*_ref_), (*x*_ref2_*, y*_ref2_*, z*_ref_), ***a*** and ***b*** are known, and there are only two unknown parameters (*t* and *s*) in Eqs. (2-1) and (2-2). For the minimum *d*^2^, the following binary equations gives *t* and *s*:

3-1 $$\partial d^{{2}} /\partial s = 0,$$

3-2 $$\partial d^{{2}} /\partial t = 0.$$

Equations (3-1) and (3-2) show that by introducing the “vector concept”, 2 muon trajectories are sufficient to locate the receiver detector if the distance [(*x*_rec2_ − *x*_rec1_)^2^ + (*y*_rec2_ − *y*_rec1_)^2^]^1/2^ is shorter than the required positioning resolution since we can approximate that the location where these two muon trajectories approach the closest is the location of the receiver such that:

4 $$\left( {x_{{\text{p}}} , \, y_{{\text{p}}} , \, z_{{\text{p}}} } \right)\sim \left( {x_{{\text{q}}} , \, y_{{\text{q}}} , \, z_{{\text{q}}} } \right)\sim \left( {x_{{{\text{rec1}}}} , \, y_{{{\text{rec1}}}} , \, z_{{{\text{rec}}}} } \right)\sim \left( {x_{{{\text{rec2}}}} , \, y_{{{\text{rec2}}}} , \, z_{{{\text{rec}}}} } \right).$$

The maximum positioning error in the *z* direction (*δz*) when defining the location of the receiver detector in this way is:

5 $$\delta z = \left( {W_{{1}}^{{2}} + W_{{2}}^{{2}} } \right)^{{{1}/{2}}} {\text{tan}}\theta *$$

where θ* is the angle formed by these 2 muon trajectories, and *W*_1_ and *W*_2_ are respectively the lengths of the longer and shorter sides of the receiver detector. An example application involving the small reference detector will be described in the subsection entitled: "Emergency rescue" within the Discussion section.

With MuWNS, it is required to derive *x*_p_*, y*_p_*, z*_p_, and *t* by solving the following four-fold equations:

6 $$L_{i}^{2} = \left( {x_{i} - x_{{\text{p}}} } \right)^{{2}} + \left( {y_{i} - y_{{\text{p}}} } \right)^{{2}} + \left( {z_{i} - z_{{\text{p}}} } \right)^{{2}} + ct^{{2}} ,$$

where *L*_*i*_, *x*_*i*_^2^, *y*_*i*_^2^, *z*_*i*_^2^ and *c* are respectively the travel distance of muons, the three-dimensional vertex points formed by the muon trajectories and the reference detectors, and the speed of light in vacuum. Therefore, at least 4 and 3 muon tracks were necessarily for three-dimensional and two-dimensional positioning, respectively. Vector muPS reduces this number to 2 and 1. Since the cosmic-ray muon flux is limited, reduction of this number contributes to speeding up the process of positioning substantially. Consequently, for three-dimensional or two-dimensional positioning, the positioning frequency (positioning time resolution) is simply improved by factors of 2 and 3, respectively.

### Positioning frequency

The positioning frequency, meaning how frequently the positioning data can be received from Vector muPS is derived by the total muon tracking frequency between the reference detector and the receiver detector. The muon tracking frequency is determined by the solid angle formed between the unit reference detector and the receiver detector as well as the thickness of the overburden located above the receiver detector. The integrated open-sky muon flux is 70 m^−2^ s^−1^sr^−1^, and the zenith-angular dependent open-sky muon intensity is approximated as follows^22^:

7 $$dI/dEd\Omega \approx 0.14E^{ - 2.7} \left[ {1/(1 + 1.1E\cos \theta /115{\text{ GeV}}) + 0.054/(1 + 1.1E\cos \theta /850\;{\text{GeV}})} \right]{\text{cm}}^{ - 2} \,{\text{s}}^{ - 1} \,{\text{sr}}^{ - 1} \,{\text{GeV}}^{ - 1}$$

where the first and second terms indicate the contribution of pions and charged kaons, respectively. *E*, *Ω*, and *θ* are respectively the energy of the muons, the solid angle, and the muon's arrival angle from zenith.

The analytical muon range of the continuous slowing down approximation (CSDA)^23^ is expressed by:

8 $$- {\text{d}}E/{\text{d}}X = a + bE,$$

where the first and second terms indicate the contribution of the ionization and radiative processes, respectively. The parameters of *a* and *b* in this equation depend on the material muons travel through. With Eq. (8), the minimum energy of the muons (*E*_C_) that can pass through the overburden can be determined and by integrating Eq. (7) from *E*_C_ to infinity, the zenith-angular dependent muon tracking frequency *ϕ* can be derived per unit area and unit solid angle:

9 $$\phi = \int\limits_{0}^{{(\Omega_{0} )}} {\int\limits_{{(E_{c} )}}^{\infty } {IdEd} \Omega } ,$$

where *Ω*_0_ is the solid angle formed between the unit reference detector and the receiver detector. The active area of the detector varies depending on the angle between the detector plane and the incident muons. If the detector plane is perpendicular to the muon's incident angle (or the muon's trajectory), the active area for muon detection will be equivalent to the detector's area. Otherwise, it will be reduced such that:

10-1 $$S_{{\text{1 REFERENCE}}} = {\text{S}}_{{0{\text{ REFERENCE}}}} {\text{sin}}\theta^{*} ,$$

10-2 $$S_{{\text{1 RECEIVER}}} = {\text{S}}_{{0{\text{ RECEIVER}}}} {\text{sin}}\Theta^{*} ,$$

where *S*_1 REFERENCE_ and *S*_1 RECEIVER_ are respectively the effective active areas of the reference detector and the receiver detector, *S*_0 REFERENCE_ and *S*_0 RECEIVER_ are respectively the detector's areas of the reference detector and the receiver detector, and θ^*^ and Θ^*^ are respectively the angle formed between the incident muon's trajectory and the reference detector plane and the receiver detector plane. Accordingly, the solid angle formed between the reference detector and the receiver detector is reduced as a function of θ^*^ and Θ^*^ for a given distance between the reference detector and the receiver detector.

### Positioning accuracy

The positioning accuracy of the receiver is determined by the angular resolution (*δθ*,* δϕ*) of the reference detector and the receiver detector. In this work, it was assumed that the angular resolutions of these detectors are the same, and the zenith angular resolution and azimuth angular resolution are the same: *δθ* = *δϕ*. The positioning accuracy (*δx*) at (*x*, *y*, *D*) is:

11-1 $$\delta x = \delta \theta \left[ {\left( {x - x_{{{\text{ref}}}} } \right)^{{2}} + \left( {y - y_{{{\text{ref}}}} } \right)^{{2}} + D^{{2}} } \right],$$

11-2 $$= \delta \theta L,$$

where *x*_ref_ and *y*_ref_ are respectively the 2-dimensional muon hitting positions, on the reference detector. As can be seen in Eq. (11-2), the positioning accuracy is proportional to the distance (*L*) between the reference detector and the receiver detector. Therefore, the measurement accuracy/resolution is highly dependent on the distance between the client and the reference. Since an angular resolution of ≤ 10 mrad for determining *θ* and *ϕ* can be easily achieved with a currently available muography detector, it is easy to attain cm-level positioning accuracy for *L* ≤ 10 m. For example, the angular resolution reported in Reference^24^ is 2.7 mrad.

### Positioning noise

It is assumed that the receiver detector's size is sufficiently smaller than the size of the reference detector. The amount of positioning noise increases/decreases according to the accidental coincidence of the independent muon events within the given solid-angular window (*δΩ*). Since muons arrive from different points throughout the entire hemisphere, incident angles of two independent muons can be accidentally measured as the same angles within a given angular window; this accidental rate can be calculated as follows. The equation for small solid angles of *δΩ* is:

12 $$\delta \Omega \sim \delta \theta \delta \phi = \theta_{{\text{W}}}^{{2}} ,$$

since the solid angle is approximated to be *δS*cos*θ D*^-2^, where cos*θ* ~ 1 for *θ* <  < 1. *θ*_W_ is the angular window used for taking an angular coincidence. Here, it was assumed that the given angular window is equivalent to the angular resolution of the detector. When a muon passes through the reference detector with an incident angle of (*θ*, *ϕ*), the muon rate (*I*_W_) within this angular window (*θ* + *θ*_W_, *ϕ* + *θ*_W_) is therefore given by:

13 $$IW = \theta W^{2} \int\limits_{0}^{2\pi } {\int\limits_{{(E_{c} )}}^{\infty } {IdEd\Omega } } ,$$

Consequently, the possibility that the muons measured with the receiver detector within this angular window accidently matches with those muons measured with the reference detector within the angular window (*δθ*) (the matching track possibility) is proportional to *δθ*^2^. For a given angular window, the noise ratio, the ratio of the accidental random positioning information (which contaminates the true positioning information) to the true positioning information, depends on the value of the verification time window (*T*_w_) between the "reference tracks" (which is determined by the reference detector) and the "receiver tracks" determined by the receiver detector. The rate (*A*_W_) of these matching tracks (accidental rate) expected within *T*_w_ is:

14 $$A_{{\text{W}}} = I_{{\text{W}}} T_{{\text{w}}} .$$

**Figure 2:**
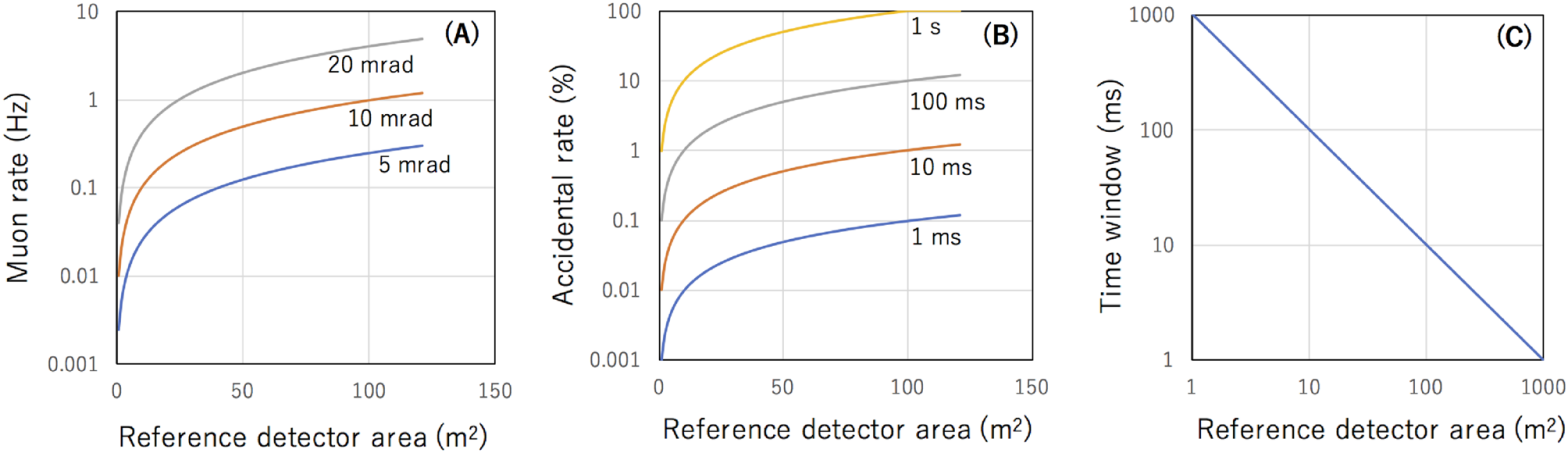
Positioning noise. The muon rate (*I*_W_) at the reference detector counted within different angular windows (*θ*_W_ = 5 mrad, 10 mrad, and 20 mrad) (**A**), the accidental rate (*A*_W_) for different time windows (*T*_w_ = 1 ms, 10 ms, 100 ms, and 1 s) (**B**), and *T*_w_ required to suppress *R*_W_ below 1% (**C**) are shown as a function of the size of the reference detector area.

For example, for *S*_0 REFERENCE_ = 10^2^ m^2^, θ^*^ = 0 rad, and *θ*_W_ = 10 mrad, *I*_W_ ~ 1 Hz, and if we set *T*_w_ = 1 ms, *A*_W_ would be ~ 10^–3^ (0.1%). As long as the receiver detector's size is sufficiently smaller than the size of the reference detector, this noise ratio is independent from the muon event rate at the receiver detector (or the size of the receiver detector). Figure 2 compares the true tracking rate and the accidental tracking rate for different *δθ* values.

### Navigation models

**Figure 3:**
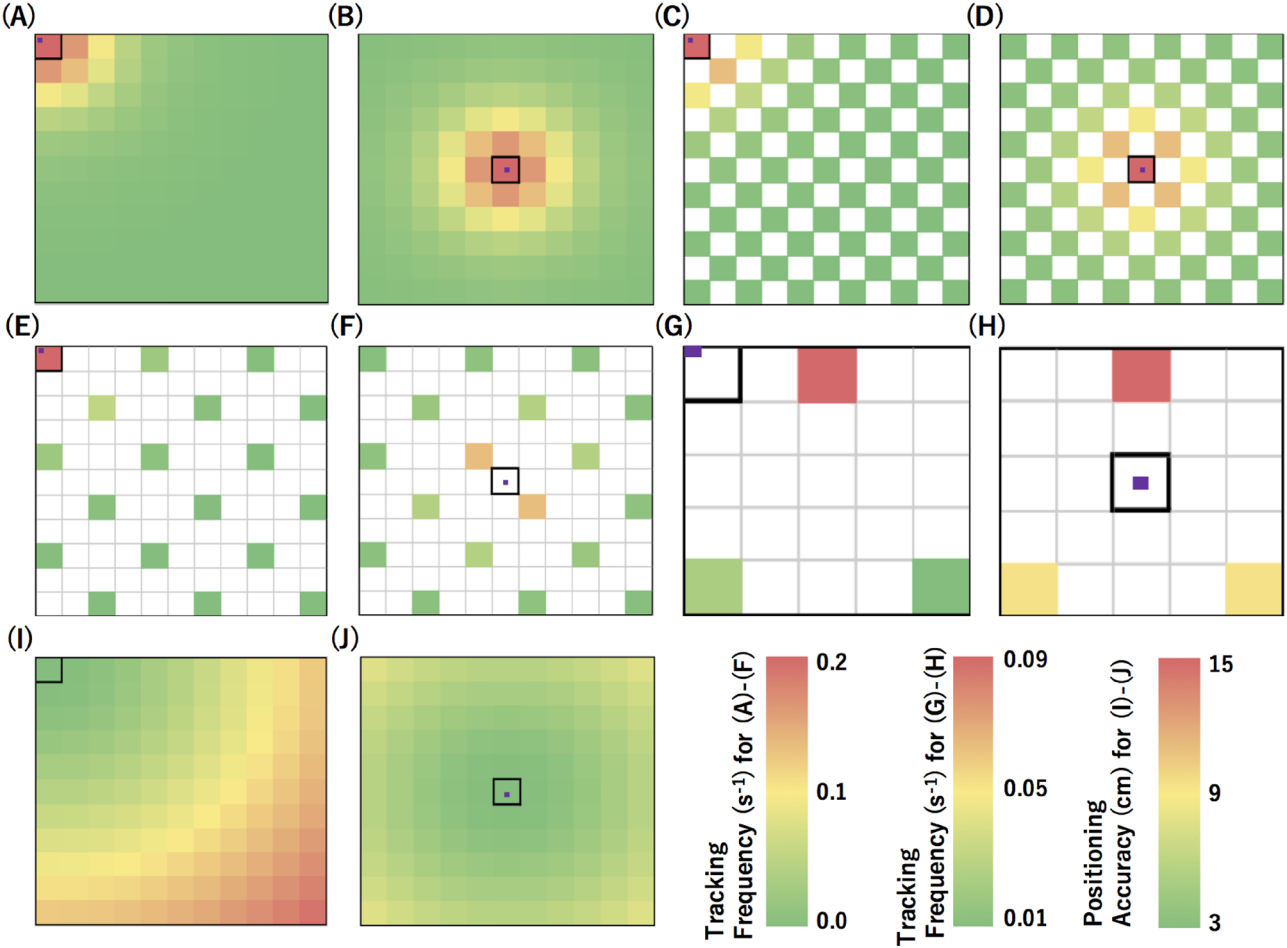
Navigation models. The solid grid boxes indicate the unit detectors with the colors ranging from green to red to represent different values of tracking frequency (for **A**–**H**) and position accuracy (**I**, **J**). Each receiver detector is represented as a smaller solid purple box surrounded by an unfilled box outlined in black. The size of the grid is 1 m × 1 m. (**A**–**F**) are drawn for a vacant room measuring 11 m × 11 m and (**G**, **H**) are drawn for a vacant room measuring 5 m × 5 m. (**I**, **J**) respectively shows the positioning accuracy based on the placement of the receiver detector.

For indoor modelling of a navigation scenario, a reinforced concrete (RC) building was assumed as a housing unit for the current simulation study. Cosmic-ray muons were generated in accordance with the open-sky muon spectrum (Eq. 7) and injected into the building. In the indoor simulations, it was assumed that no materials existed above the roof, and the thickness of the roof (30 cm concrete) was disregarded since *E*_C_ for 30-cm thick SiO_2_ is less than 200 MeV^23^. The top left corner (P1) and the center (P2) (marked in Fig. 3 with filled, purple rectangles in unfilled, black outlined squares) are respectively the least favorable and most favorable tracking rate locations in this space and were chosen as the positions for the receiver detector in this model. For underground simulations, only the muons with energies above *E*_C_ were injected to the room. The overburden was assumed to be made of SiO_2_ with a bulk density of 2 g cm^-3^. The parameters, *a* and *b* in Eq. (8), have been taken from Groom et al.^23^ It was assumed that the receiver detector was located at a height of 2 m from the floor (as an analogy for navigation of forklifts in a factory building where the receiver detector is placed on the roof of these forklift). The reference detector and the receiver detector were both placed in positions which were horizontal to the ground surface, and the tracking resolution of these detectors were assumed to be 10 mrad. The following two cases are considered, each having different sized rooms:

(A) *A room measuring 11 *m* in length* × *11 *m* in width.* In this model, the reference detector consists of smaller detector components, each having a unit size of 1 × 1 m^2^ and each referred to as a “unit detector”. The reference detector is placed right beneath the ceiling of this room. There were three different configurations of reference detectors which were examined with different numbers of unit detectors used as the components of the reference detector: (A-1) 121 unit detectors, (A-2) 61 unit detectors, and (A-3) 15 unit detectors. The geometrical configurations of the unit detectors are shown for these patterns in Fig. 3A–F. Each unit detector configuration, (A-1), (A-2), (A-3), has 2 variations based on where the receiver detector was placed. (A-1) corresponds to Fig. 3A, B, (A-2) corresponds to Fig. 3C, D, and (A-3) corresponds to Fig. 3E, F. The size of the receiver detector was assumed to be the size of an iPad (250.6 × 174.1 mm^2^).
(B) *A room measuring 5 *m* in length* × *5 *m* in width.* In this model, the reference detector was also placed right underneath the ceiling of this room, and the unit size of the reference detector and size of the receiver detector were the same as (A), i.e., 1 × 1 m^2^ and 250.6 × 174.1 mm^2^, respectively. However, only 3 unit detectors were used for the reference detectors in this scenario. The geometric configuration of the reference detector is shown in Fig. 3G, H.

Figures 3A–H show the tracking rate expected from the different combinations between the receiver detector and the unit detectors used as the components of the reference detector. The positioning frequency is therefore the integration of the tracking rate for all of the unit detectors. Using Eq. (9), the positioning frequency (*Φ*) attained by the reference detector that consists of a *N*_*x*_ × *N*_*y*_ matrix of the unit detectors is given by:

16 $$\Phi = \mathop \sum \limits_{j = 1}^{{N_{y} }} \mathop \sum \limits_{i = 1}^{{N_{x} }} \phi_{i,j}$$

where *ϕ*_*i,j*_ is the muon tracking frequencies measured between the receiver detector and the unit detectors located at the geometrical address (*i*, *j*). The averaged positioning accuracy < *δx* > is then given by:

17 $$\left\langle {\delta x} \right\rangle = \Phi^{ - 1} \mathop \sum \limits_{j = 1}^{{N_{y} }} \mathop \sum \limits_{i = 1}^{{N_{x} }} x_{i,j} \Phi_{i,j} .$$

As can be seen in Fig. 3A–H, while the flux contribution is at its maximum at the point corresponding to the component of the receiver detector located nearest to the reference detector, even the muons that travel a longer distance between the reference detector and the receiver detector can be used for positioning. On the other hand, as shown in Eq. (11-2) and Fig. 3I, J, the positioning accuracy is degraded when the muons are tracked by unit detectors located further away. However, the receiver-unit tracking rate is significantly reduced as a function of the distance between the receiver detector and the unit detector (Fig. 3A–H). This tradeoff determines the positioning accuracy. The positioning frequency and the positioning accuracy were studied for these 12 patterns, and the results will be described in the next subsection.

### Simulation results

Tables 1 and 2 summarize the simulation results for a ceiling height of 5 m. While Table 1 shows the navigation results without an overburden, Table 2 summarizes the results with an overburden with a thickness of 1.5 m. This thickness corresponds to the total floor thickness of a typical 10-story RC building. The following three features can be exploited from these tables.

(A) There is no large difference in the positioning frequency and the averaged positioning accuracy between the room without an overburden and the room with an overburden. This is because *E*_C_ is ~ 600 MeV for 1.5-m concrete^23^ which is below the peak energy of the differential spectrum of cosmic-ray muons^22^.
(B) The averaged positioning accuracy is slightly higher in the room with an overburden. As was described in the previous subsection, the positioning accuracy is degraded by the contribution of the muons tracked by unit detectors located further away from the reference detector. However, when an overburden exists above the receiver detector, this contribution is reduced since the muons arriving at more horizontal angles will be more attenuated on average than the muons arriving at more vertical angles.
(C) The averaged positioning accuracy doesn't depend on the number of the unit detectors used for the receiver detector. While the positioning frequency is reduced in proportion to the total area of the reference detector, it doesn't significantly depend on the number of the unit detectors used for the reference detector.

**Table 1:** Navigation results without an overburden.

	Positioning frequency (Hz)		Averaged positioning accuracy (cm)	
	(P1)	(P2)	(P1)	(P2)
(A-1)	1.79	4.42	4.48	4.17
(A-2)	0.92	2.22	4.44	4.18
(A-3)	0.33	0.57	4.07	4.27
(B)	0.13	0.20	4.09	3.88

P1 and P2 are respectively the positions at the top left corner and the center (marked in Fig. 3 with filled, purple rectangles inside unfilled, black outlined squares).

**Table 2:** Navigation results with an overburden.

	Positioning frequency (Hz)		Averaged positioning accuracy (cm)	
	(P1)	(P2)	(P1)	(P2)
(A-1)	1.60	4.06	4.22	4.10
(A-2)	0.83	2.03	4.18	4.10
(A-3)	0.31	0.52	3.84	4.18
(B)	0.12	0.19	4.03	3.88

P1 and P2 are respectively the positions at the top left corner and the center (marked in Fig. 3 with filled, purple rectangles inside unfilled, black outlined squares).

Consequently, we can change the number of unit detectors used for the reference detector in accordance with the time resolution required for indoor navigation without degrading the positioning accuracy. In conclusion, the number of the unit detectors is a parameter which should be chosen according to how frequently the user would like to receive the positioning data; this usually depends on the speed of the object to be navigated.

**Figure 4:**
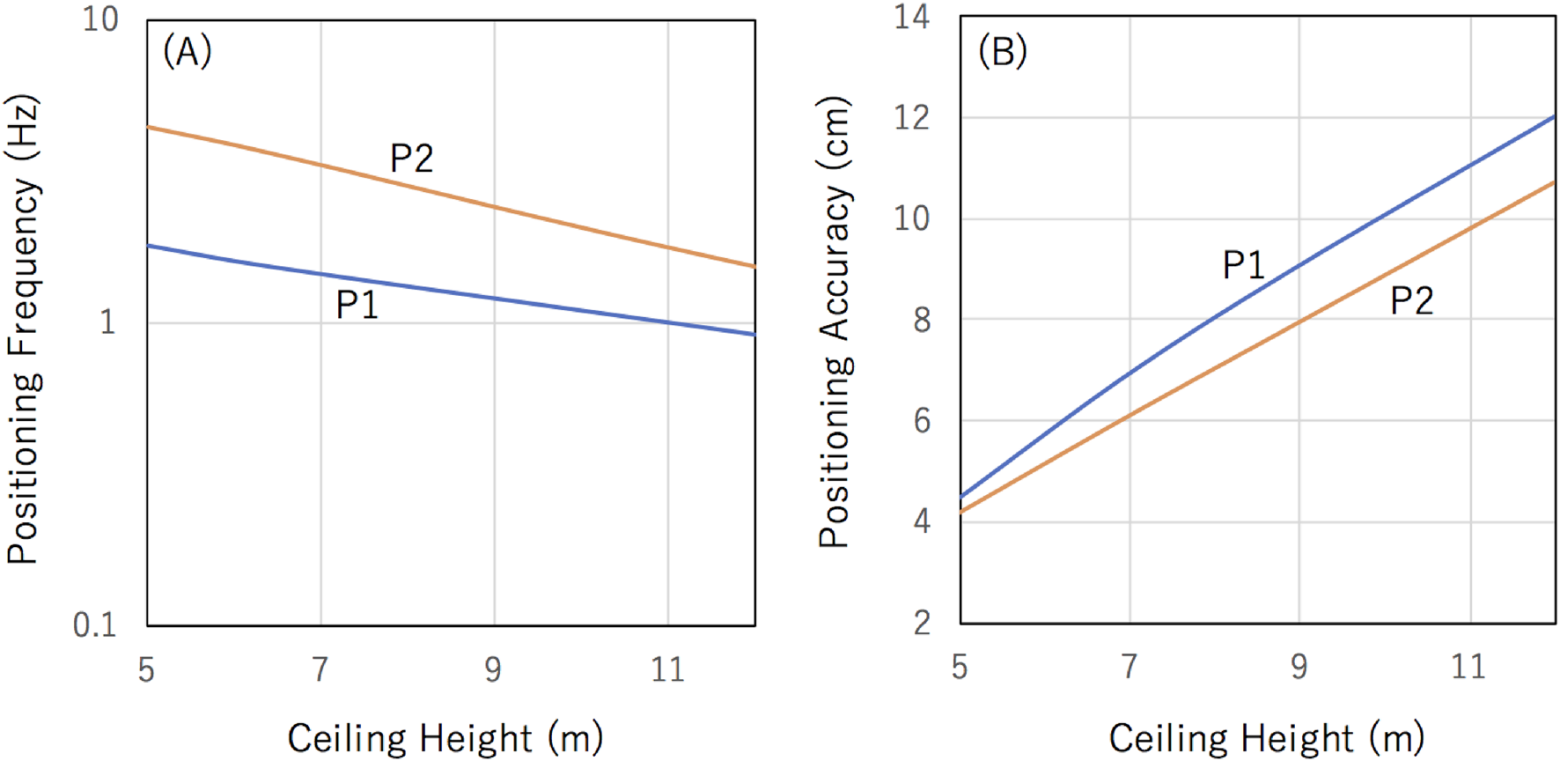
Ceiling height effect. The positioning frequencies (**A**) and the positioning accuracies (**B**) are shown as a function of the ceiling height.

Figure 4 shows the positioning frequencies and the positioning accuracies measured at the locations P1 and P2 as a function of the ceiling height. The number of the unit detectors was assumed to be 121, but the trend is similar for other unit detector configurations: (A-2) and (A-3). While there is a general trend that the positioning frequency is reduced as a function of the ceiling height, the reduction speed is slower at P1 than that at P2 (Fig. 4A). The primary reason of this reduction in the positioning frequency is an increase in the distance (*L*) between the reference and the receiver as the ceiling height increases. However, at P1, a larger fraction of further unit detectors is used for positioning. Therefore, during the course of the averaging process shown in Eq. (17), variations in the positioning frequency at P1 are more affected by the factors of (*x* − *x*_ref_)^2^ + (*y* − *y*_ref_)^2^ in *L*. If the size of the reference detector is unlimited (or sufficiently larger than the navigation area size), the ceiling height dependences in the positioning frequency are supposed to be the same at P1 and P2. However, in reality, the size of the reference detector is limited, and consequently, if the client position is different, the positioning frequency (the muPS signal update rate) will not be reduced in the same way as a function of the ceiling height. Consequently, as can be seen in Fig. 4B, degradation in the positioning accuracy at P2 is smaller than that at P1. There are less factors to degrade the positioning accuracy at P2 since the receiver detector at P2 collects less muons that passed through further unit detectors than that at P1.

## Discussion

The goal of Vector muPS is the realization of RTK-GPS level navigation capability in indoor environments. With typical RTK-GPS devices, the positioning accuracy of a few cm is attainable after a few minutes of initial data taking. After the first few minutes, the devices output the positioning information every second, while maintaining this positioning accuracy. Tables 1 and 2 show that the equivalent of RTK-GPS level navigation capability is attainable with Vector muPS for areal coverage ratios (100%, 50% and 12%) of the reference detector. In the following section, techniques which work well in tandem with Vector muPS and possible application cases are discussed.

### Vector muPS-INS solution

The Vector muPS's signal update rate is not sufficient for the purpose of high-speed object navigation. If this kind of navigation is necessary, combining it with another technique might be a good solution to realize seamless positioning. Since the objective of muPS is navigation that isn't affected by physical obstacles in the environment, the additional technique selected for this integrated system should be a system which does not use signals sourced outside of the receiver; LiDAR, sonar, and Bluetooth are examples of systems with signals outside the receiver. To address this, the combination of INS and Vector muPS is ideal. Although the Vector muPS signal update rate (which is close to the GPS signal update rate) is less when compared to INS, since the INS positioning error grows as a function of time, this drift can be repeatedly calibrated with an aid of Vector muPS through online calibration of INS^25–27^. INS has been already used for GPS-based navigation during periods when GPS signals are interrupted^27–29^. Unlike GPS, muPS signals will never be interrupted, INS can be more reliably used to operate the system with a less drift effect.

### Angular configuration control

A cm-level positioning accuracy is attainable with Vector muPS as long as the angular configuration between the reference detector and the receiver detector is well defined. However, in general, the angles determining the receiver detectors’ active areas tend to change (incline) as the receiver detector moves further away from the reference detector, and the level of this inclination has to be accurately monitored. This is a drawback of Vector muPS in comparison to conventional MuWNS (which utilizes the TOF information between the reference detectors and the receiver detector). On the other hand, if *θ*_W_ = 10 mrad, then only a 1-mrad (1‰) accuracy would be sufficient for this inclination monitoring. By utilizing a dedicated inclinometer, achieving this level of accuracy is easy. For example, the Digi-Pas 2-Axis Ultra Precision Inclinometer (DWL8500XY) can achieve an accuracy of ± 1 arcsec (± 5 × 10^–6^ rad) at 0 to ± 1080 arcsec (± 5 mrad); ± 3 arcsec (± 1.5 × 10^–5^ rad) at other angles^30^. This level of accuracy is sufficient for operation of Vector MuWNS. As a matter of fact, a 1-mrad accuracy is attainable even with recent smartphone-based inclinometers which can measure changes in movement with up to 1.7 × 10^–4^ rad precision)^31^.

The most attractive feature of Vector muPS in comparison to conventional MuWNS is that a precise clock is not required to attain cm-level positioning accuracy. Clocks with a size and accuracy appropriate to MuWNS are usually expensive. For example, in order to respond to the social demands of the 5G era, the development of the chip scale atomic clock (CSAC), which is compatible with MuWNS, has been accelerated. The currently commercially available CSAC is Microchip SA65 CSAC^32^ and its short-term stabilities (1 SD) are respectively 300 ps, 1 ns, and 3 ns after 1 s, 10 s, and 100 s elapses. However, this specification indicates that positioning accuracy better than 1 m is not attainable with CSAC if the positioning frequency is lower than 0.1 Hz. Also, the cost per chip is rather high ($5,756.75 USD)^33^.

### Timing scheme

Unlike MuWNS, Vector muPS doesn't require sub-nanosecond timing information for positioning. However, a moderate time synchronization accuracy (1 ms^−1^ s depending on the total size of the reference detector) is required for verification of the muon events (Fig. 2C). This level of time synchronization accuracy is easily achievable with WiFi-based time synchronization devices such as Wi-Fi Alliance Wi-Fi CERTIFIED TimeSync^34^ or even with commercially available and low-priced quartz clocks. A commercially available generic quartz-based clock typically drifts ~  ± 20 s month^-1^ which is tolerable for operations ranging from a few hours to one day.

Since extreme time resolution is not required for the Vector muPS detectors, receiver detectors could be compacted to a hand-held scale. MINIPIX^35^, for example, is a commercially-available position sensitive detector with a matrix of 256 pixels by 256 pixels. Since the size of each pixel is 55 µm, 10-mrad angular resolution can be achieved within the thickness of 5.5 mm. The readout electronics unit is incorporated within the MINIPIX USB device. Since the dimension and the weight of this MINIPIX USB device are respectively 88.9 × 21 × 10 mm and 30 g, it can be easily incorporated into smartphones. While 5 V of electricity is applied to the detector, 500 mA current flows at the maximum (only when an event is detected), therefore the power consumption is more than one order of magnitude lower than 2.5 W in low radiation environments^36^. In the following subsections, several example applications of Vector muPS will be discussed including three scenarios which would benefit from using a hand-held scale detector.

### Factory automation

**Figure 5:**
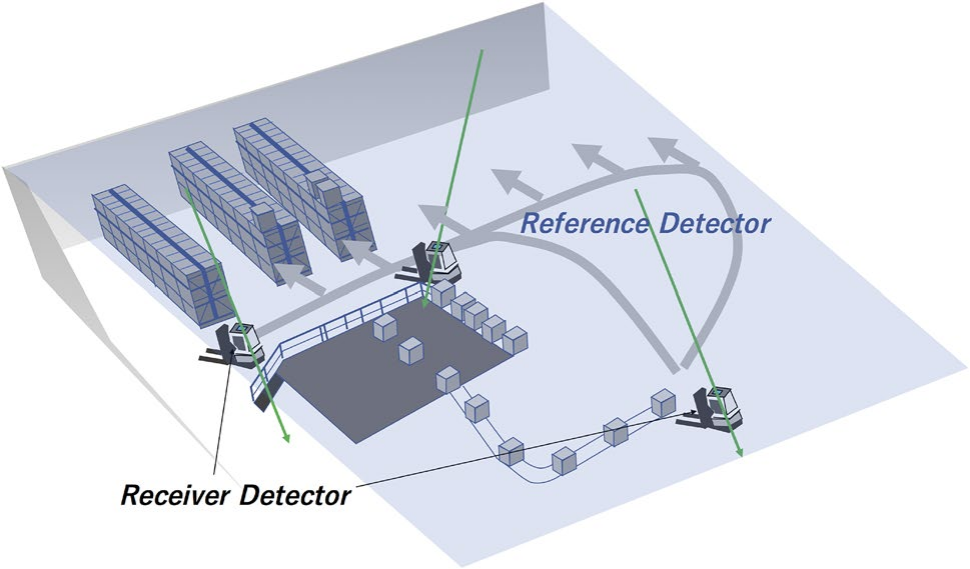
Vector-MuWNS-based factory automation scheme. Green arrows indicate muon trajectories. Gray arrows indicate an example of the programmed routes of the forklifts. In this scheme, the ceiling of the room (blue translucent plane labeled with the blue text “Reference Detector”) is assumed to be the location of the reference detector. In this case, the ceiling is fully covered with the reference detector.

In this scenario, we assume a forklift which moves exclusively on the same *X*–*Y* plane to transport materials to and from various locations (Fig. 5). The Vector muPS reference and receiver detectors could be either MWPC-based detectors^24^ or scintillator-based detectors^37^. An angular resolution of 10 mrad is easily attained by these types of detectors. In factories, Wi-Fi CERTIFIED TimeSync or similar systems can be used for communication between the reference detector and the receiver detector. The current cost required for making reference detectors including the readout electronics is ~ $3 k USD m^-2^. Therefore, the total cost required for building up the Factory navigation system is ~ $3 k USD m^−2^, ~ $1.5 k USD m^−2^, and ~ $450 USD m^−2^ respectively for areal coverage ratios (100%, 50% and 12%) of the reference detector. However, if mass produced, this cost could be substantially reduced.

### Emergency rescue

When landslides and building-collapses are caused by large-scale natural disasters such as heavy rains and earthquakes, sometimes people are confined inside a collapsed building or buried underneath rubble. In many cases, they cannot move even if they want to call for help. GPS installed in smartphones are helpful tools to locate people in many cases. However, it becomes very difficult to pinpoint a position in the rubble where the GPS signal is blocked. Moreover, if rescuers walk on top of rubble to pinpoint the location of trapped individuals, there is a risk that at unseen weak spots in the rubble, their weight will cause further collapses, possibly causing a secondary disaster. Therefore, exploration methods using drones are being developed. With this method, a drone is equipped with a highly directional antenna, and it emits radio waves that reach deep into rubble, enabling communication between the drone and a mobile phone^38^. The signal strength received by the mobile phone is used for locating this mobile phone. Although this method enables rescue drone operators to roughly estimate the horizontal location of the mobile phone, the vertical location of the mobile phone cannot be derived. Vector muPS will solve this problem. By installing a muon tracking chip (like the commercially available MiniPIX) in a mobile phone, it is expected that the three-dimensional position of a "person in need of rescue" can be determined with cm-level accuracy.

**Figure 6:**
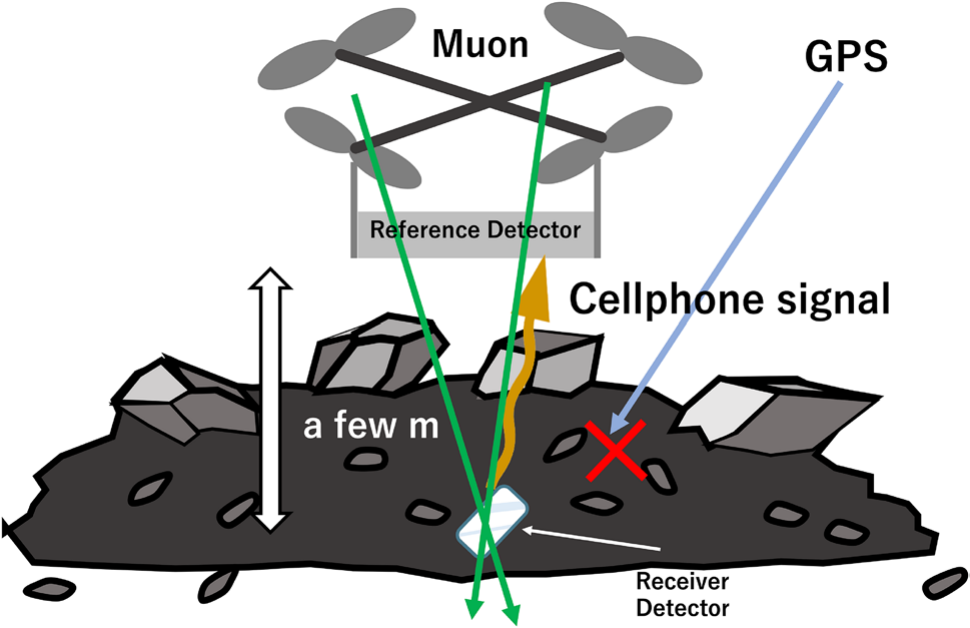
Drone-Vector-Mu-WNS emergency rescue scheme. The copyright of this image is owned by HKMT.

The drone-based procedure to apply Vector muPS (Fig. 6) to locating trapped individuals after a disaster is as follows.

(A) Mount a muon tracker on a drone.
(B) A drone-based search is performed by emitting radio waves towards the ground.
(C) Direct the drone to hover at the point where the strongest radio wave was detected.
(D) Receive 3D positional information from the muon tracking chip inside the mobile phone.

In (C) and (D), the time required for receiving the three-dimensional position information of the muon tracking chip is the time required to record at least two muon tracks. For example, if the area of the muon tracker mounted on the drone is 1 m^2^ and MiniPIX installed in a smartphone is used as the muon tracking chip (the sensitive area of 2 cm^2^), the time required to record two muon tracks is 1 min when the drone is located 1 m above the MiniPIX device and 4 min when the drone is located 2 m above the MiniPIX device. This capability may become game-changer in the future for search and rescue teams responding to a wide range of emergency situations.

In conclusion, Vector muPS enables cm-level positioning in indoor environments within a reasonable time scale. MuWNS systems have been designed to be virtually unaffected by obstacles located within the navigation range (such as steel-made separations, platforms, racks, etc.). However, the Vector muPS concept is more flexible than MuWNS since the minimum unit system required for positioning can consist of only two muon trackers without the necessity of additional devices (such as local clocks) that can potentially degrade stability of navigation, accuracy of navigation, and can increase the total cost of the system. Although the region which can be navigated is limited only to areas underneath the reference detectors, as described in this paper, the unique capacities of Vector muPS (used alone or in tandem with other techniques) has a great potential to contribute to several improvements for numerous industrial and societal applications.

## Data Availability

The datasets used and/or analyzed during the current study are available from the corresponding author on reasonable request.
